# Isolation of Oxyberberine and *β*-Sitosterol from *Berberis lycium* Royle Root Bark Extract and *In Vitro* Cytotoxicity against Liver and Lung Cancer Cell Lines

**DOI:** 10.1155/2020/2596082

**Published:** 2020-06-15

**Authors:** Muhammad Asad Anwar, Shahzadi Tabassam, Muhammad Gulfraz, Muhammad Sheeraz Ahmad, Ghazala Kaukab Raja, Muhammad Arshad

**Affiliations:** ^1^University Institute of Biochemistry and Biotechnology, Pir Mehr Ali Shah Arid Agriculture University, Rawalpindi 46300, Pakistan; ^2^Department of Botany, Pir Mehr Ali Shah Arid Agriculture University, Rawalpindi 46300, Pakistan

## Abstract

*Berberis lycium* Royle has been traditionally used to cure rheumatism, eye and ear diseases, malarial fever, diabetes, stomach disorders, and skin diseases. There is a least amount of data available on cytotoxic capacity of *Berberis lycium* from Pakistani origin, so on this basis, the present study was aimed to screen *Berberis lycium* root bark extracts for cytotoxicity against cancer cell lines and isolation of chemical constituents from the most cytotoxic extract. Initial screening of extracts was performed on HepG2 cells at 100 *μ*g/mL for 72 hours of treatment by using an MTT assay. Active fractions were subjected to a series of column chromatographies for the isolation of cytotoxic compounds. Molecular structures were elucidated by using combined data from ^1^H-NMR, ^13^C-NMR, and ESI-MS graphs. Assessment of reduction in cell proliferation by isolated compounds was performed on three human cancer cell lines (SK-Hep-1, HepG2, and NCI-H1299). Both *n*-hexane and chloroform fractions were found active with percent cell viabilities of 8.41 ± 2.23 and 22.31 ± 9.11 in HepG2 cells compared with lupeol 35.43 ± 3.35 percent viability. A protoberberine alkaloid identified as oxyberberine was isolated from chloroform fraction while *β*-sitosterol was isolated from *n*-hexane fraction. Oxyberberine inhibited SK-Hep-1 cell proliferation under a dose-dependent manner with an IC_50_ value of 34.26 ± 3.34 *μ*M while HepG2 cells showed 50% inhibition at 62.96 ± 4.12 *μ*M. *β*-Sitosterol showed reduction in cell viability in SK-Hep-1 cells and HepG2 cells with IC_50_ values of 123.12 ± 3.51 *μ*M and 140 ± 4.21 *μ*M. This is the first report on the isolation of oxyberberine and *β*-sitosterol from *Berberis lycium* root bark and their cytotoxic evaluation against SK-Hep-1 and NCI-H1299 cells. The cytotoxic potential of *Berberis lycium* Royle extracts and isolated compounds is suggesting that it is a promising candidate for anticancer drug discovery.

## 1. Introduction

Cancer is the abnormal mass of cells that divide without control. It is the second driving reason for death around the world. Approximately, 9.6 million deaths in 2018 are reported due to cancer globally (the WHO). Besides ageing, UV radiations, infections from viruses, and tobacco smoke are the major causes of cancer [1]. There are different methods to treat cancer, which include radiotherapy, chemotherapy, and surgery. A type of treatment in which cancer cells are killed or growth of cancer cells is retarded by using chemicals is categorized as chemotherapy. It is a successful method to treat different cancers besides radiotherapy and surgery [2]. A number of findings demonstrated that the cytotoxic assessment of plant extracts or isolated compounds has proven a way to develop therapeutics that target rapidly dividing cancer cells. Plants have given numerous viable anticancer agents which are in current use, for example, vinblastine, irinotecan, topotecan, vincristine, and taxanes [3]. Cytotoxicity screening models give significant information to pin point plants with potential cytotoxic effects [4]. In spite of extensive research on exploration of new cytotoxic agents, there are numerous plant species yet to be investigated, and *Berberis lycium* Royle is one of them.

*Berberis lycium* Royle belongs to a major dicotyledonous genus *Berberis* and family Berberidaceae [5]. It is a spiny evergreen shrub with characteristic yellow wood, found growing in hilly areas (1400 m–3500 m above sea level) of Pakistan [6]. It is used as food as well as the key component of many herbal preparations used to treat rheumatism, eye and ear diseases, malarial fever, diabetes, jaundice, stomach disorders, and skin diseases [7] The reported biological activities of *Berberis lycium* Royle include antioxidant [8], antidiabetic [9], anticancer [10], wound healing [11], and antimicrobial [12]. Qualitative analysis of phytochemicals indicate the presence of many classes of compounds including alkaloids, saponins, cardioactive glycosides, hydrolysable tannins, phytic acid, vitamin C, and *β*-carotene in the plant. Alkaloids serve as a rich source for anticancer drug discovery. Many plant-based alkaloids have been successfully developed as anticancer drugs including vinblastine, vinorelbine, vincristine, and vindesine. The main type of alkaloid present in *Berberis* is protoberberine alkaloids. The reported bioactive alkaloid with cytotoxic activity is berberine [13].

*Berberis* species from Pakistan are not well investigated for cytotoxic potential, and there is a least amount of data available on cytotoxicity on human cell culture systems [6]. Hence, the present study was aimed to screen *Berberis lycium* root bark extracts for cytotoxicity against cancer cell lines and isolation of chemical constituents from the most cytotoxic extract.

## 2. Materials and Methods

### 2.1. Instruments and Materials

Nuclear magnetic resonance spectroscopy was done on a Bruker Ascend 400 spectrometer. A Bruker Bio TOF IIIQ (quadrupole time-of-flight) mass spectrometer was used for the determination of molecular mass. Silica gel for column chromatography and for TLC (10–40 *μ*m, GF254) was obtained from Qingdao Haiyang Chemical Co., Ltd. (QHCC), China. Precoated TLC was developed by using various solvents, and developed plates were observed at 254 or 365 nm under a UV lamp and sprayed with Dragendorff's reagent for alkaloids, or 5% phosphomolybdic acid solution for steroids and terpenoids, or placed in a jar saturated with iodine vapours for the detection of phenolic steroids and fatty acids to make spots visible. Commercial grade solvents were purchased and purified in a rotator evaporator (Heidolph, Germany) under reduced pressure prior to use. The absorbance of the formazan product formed in the MTT assay was measured on a Thermo Scientific Varioskan Flash multimode reader.

### 2.2. Plant Material

The dried root bark of *Berberis lycium* Royle was purchased from a local market situated in Ghalla Mandi near Raja Bazar, Rawalpindi, Pakistan, and identified by an expert taxonomist Dr. Muhammad Arshad, Department of Botany, Pir Mehr Ali Shah Arid Agriculture University, Rawalpindi, Pakistan.

### 2.3. Extraction and Fractionation

The desiccated and powdered root bark of *Berberis lycium* (10 kg) was soaked in methanol (15 L) at 30°C for 72 hours, filtered, and concentrated into crude methanolic extract. The crude extract (329.88 g) was partitioned into *Berberis lycium n*-hexane fraction (BLNH), *Berberis lycium* chloroform fraction (BLCH), *Berberis lycium* ethyl acetate fraction (BLEA), and *Berberis lycium* butanolic fraction (BLBU) by solvent-solvent fractionation. All the fractions were kept at 4°C for further processing [14].

### 2.4. MTT Cell Viability Assay

Extracts were evaluated for their cytotoxic effect on the basis of the MTT (3-[4, 5-dimethylthiazol-2-yl]-2, 5 diphenyltetrazolium bromide) assay on the human liver cancer cell line (HepG2) [15].

### 2.5. Cell Culture

HepG2 cells were grown in a CO_2_ incubator at 37°C. The medium used for culturing of cells was RPMI 1640 in which 10% FBS and 1% penicillin/streptomycin sulfate were added.

### 2.6. Procedure

The MTT assay is a calorimetric assay frequently utilized to check the cell metabolic activity. A yellow-colored dye is converted to purple formazan in living cells. Intensity of formazan is proportional to the living cells. The method provided by [15] was used with minor modification. Cancer cells were cultured in the 96 well with the density of 20000 cells/mL and allowed to grow for 24 hours. Various concentrations of extracts/compounds were added into wells and placed in a CO_2_ incubator at a concentration of 5% for 72 hours using DMSO (0.1%) as negative control. Then, MTT solution (10 *μ*l, 5% w/v) was introduced into each well, and the plate was placed in an incubator for 4 hours at 37°C. The contents of each well were removed and mixed with DMSO (100 *μ*L). The absorbance of the formazan product was measured at 570 nm by using a Thermo Scientific Varioskan Flash multimode reader. The experiment was performed in triplicate.

### 2.7. Isolation of Compounds

The CHCl_3_ fraction (41.3 g) was fractionated through a column packed with normal phase silica gel (100–200 mesh, 5 kg) using petroleum ether and ethyl acetate (100 : 0–100 : 0) as a solvent system. A total of 17 fractions were obtained and labeled as 1–17. Fraction 5 (1.34 g) was further fractionated through a column (5 × 55 cm) packed with 200 g of normal phase silica gel and eluting with combination of petroleum ether and ethyl acetate (85 : 15–85 : 15) to afford fractions 5.1–5.9. Compound **1** was purified from fraction 5.9 by the process of crystallization. Fraction 5.9 was (500 mg) dissolved in chloroform and methanol in 1 : 1 ratio. The solution was left undisturbed in a fume hood for slow evaporation of solvents for several days. Crystal formation was started after three days and continued to increase. After seven days, crystals were recovered from mother solution and washed thrice. These crystals were again dissolved in chloroform and methanol in 1 : 1 ratio for reverse phase TLC.

The *n*-hexane fraction (50.5 g) was fractionated through the column packed with normal phase silica gel and eluted with combination of petroleum ether and ethyl acetate (100 : 0–0 : 100). A total of 14 fractions were obtained and were labeled as 1–14. Fraction 5 (2.3 g) was fractionated through the column packed with normal phase silica gel and eluted with combination of CHCl_3_ and MeOH (100 : 0, 99 : 1, 97 : 3, 95 : 5, and 90 : 10). A total of five fractions were obtained and labeled as 5.1–5.5. Fraction 5.4 provided compound **2** (350 mg) as white powder.

### 2.8. Structure Elucidation

^1^H- and ^13^C-NMR spectra of compounds **1** and **2** were recorded in CDCl_3_. The chemical shift (d) values are given in ppm with TMS as internal standard, and coupling constants (J) are in Hz.

### 2.9. Cytotoxicity of Isolated Compounds

Cytotoxicity of isolated compounds was tested on SK-Hep-1 cells (hepatocellular carcinoma cell line), HepG2 cells (human liver cancer cell line), and NCI-H1299 cells (human non-small cell lung cancer cell line) by using the MTT cell viability assay [15].

### 2.10. Statistical Analysis

Cytotoxicity experiments were performed in triplicate, and results are expressed as mean ± standard deviation (SD). Data were analyzed with Student's-*t* test using GraphPad Prism 6.0 software. The values ^*∗*^(*P* < 0.01) and ^*∗∗*^(*P* < 0.001) were regarded as significantly different from the corresponding control group.

## 3. Results

### 3.1. Cytotoxicity of the Crude Extract and Organic Fractions

**Figure 1:**
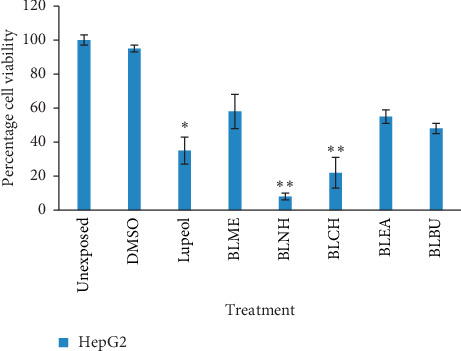
Percent viability of HepG2 cells treated with DMSO (0.1%) and 100 *μ*g/mL of different fractions of *Berberis lycium.* Cultures were exposed to treatment for 72 hours, and relative percent viabilities (mean ± SD) were measured using the MTT assay. ^*∗*^(*P* < 0.01) and ^*∗∗*^(*P* < 0.001) significantly different from unexposed cells (Student's *t*-test). BLME = *Berberis lycium* methanolic fraction, BLEA = *Berberis lycium* ethyl acetate fraction, BLNH = *Berberis lycium* n-hexane fraction, BLCH = *Berberis lycium* chloroform fraction, and BLBU = *Berberis lycium* butanolic fraction.

**Figure 2:**
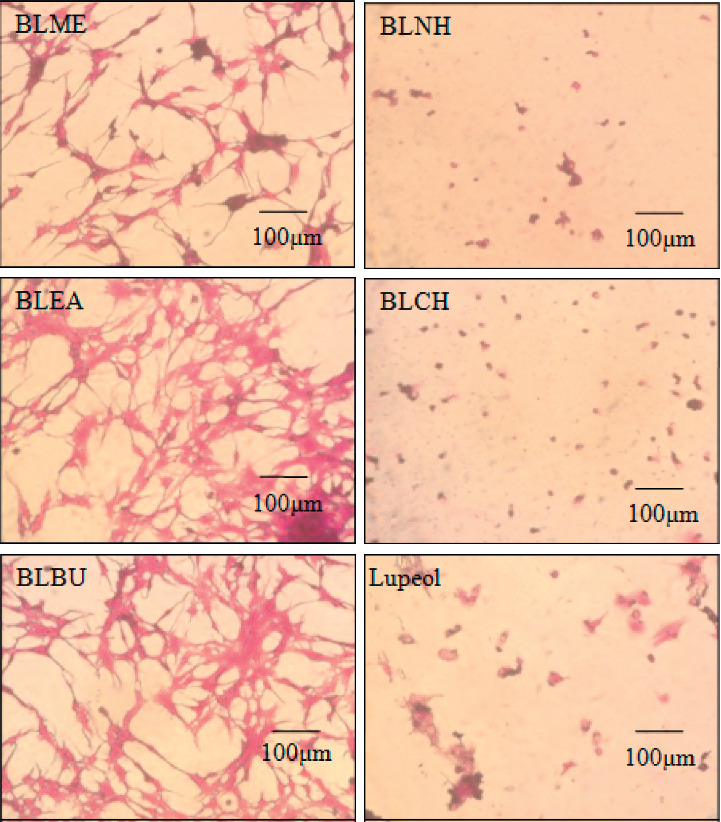
Treatment of HepG2 cells for 72 hours with 100 *μ*g/mL of different fractions of *Berberis lycium* using the MTT assay. BLME = *Berberis lycium* methanolic fraction, BLEA = *Berberis lycium* ethyl acetate fraction, BLNH = *Berberis lycium* n-hexane fraction, BLCH = *Berberis lycium* chloroform fraction, and BLBU = *Berberis lycium* butanolic fraction.

Assessment of cytotoxicity of extracts and fractions was done by calculating percent cell viability at 72-hour cultures (*n* = 3, mean ± SD), as shown in Figures 1 and 2. *Berberis lycium* n-hexane (BLNH) fraction exhibited the highest reduction in cell proliferation with a percent cell viability of 8.41 ± 2.23 at 100 *μ*g/mL. *Berberis lycium* chloroform fraction (BLCH) occupied the second position in reduction in cell proliferation with a percent cell viability of 22.31 ± 9.11. *Berberis lycium* butanolic fraction (BLBU), ethyl acetate fraction (BLEA), and methanolic fraction (BLME) showed mild reduction in cell proliferation with percent cell viabilities of 48.17 ± 2.41, 55.41 ± 1.83, and 58.17 ± 4.19, respectively. Lupeol was used as a standard drug for comparison with the results of fractions. It showed reduction in proliferation with a percent cell viability of 35.43 ± 3.35 in cells while DMSO showed 95.04 ± 3.29. The unexposed sample showed 100% cell viability. Both *Berberis lycium n*-hexane fraction (BLNH) and *Berberis lycium* chloroform fraction (BLCH) were found significantly different from the unexposed sample at *P* < 0.001.

### 3.2. Isolation and Characterization of Compounds

Both *n*-hexane and chloroform fractions were subjected to repeated column chromatography, and two compounds were isolated. Compound **1** was obtained as yellow crystals, and when this is spotted on TLC and sprayed with Dragendorff's reagent, they give orange color that indicates alkaloids. The compound gave the molecular ion peak at m/z 351 as the base peak in the El-MS spectrum. The combined information from ^1^H- and ^13^C-NMR concluded that compound **1** is an 8-oxo-protoberberine derivative [16].

**Figure 3:**
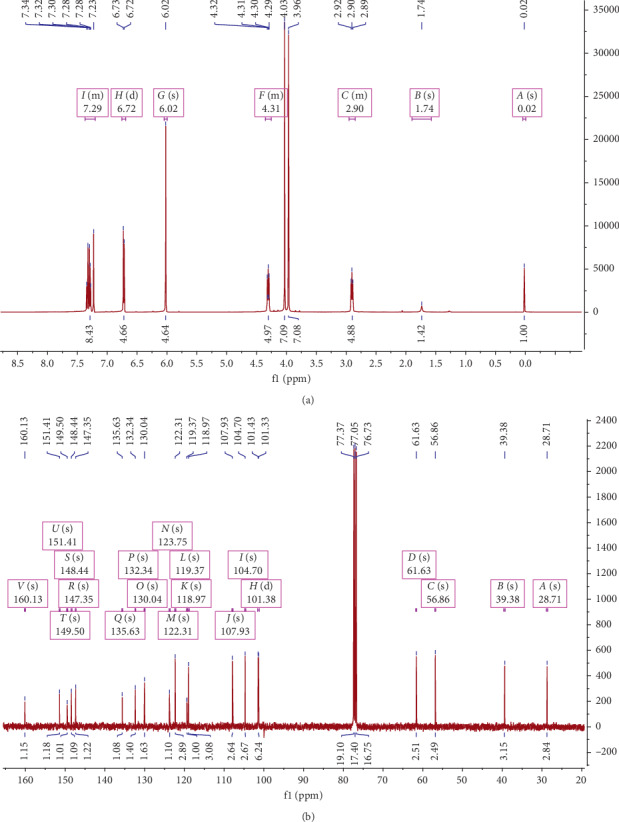
^1^H-NMR (a) and ^13^C-NMR (b) spectra of oxyberberine recorded in CDCl_3_. The chemical shift (d) values are given in ppm with TMS as internal standard, and coupling constants (J) are in Hz.

Skeleton of protoberberine is provided by various signals in proton NMR spectra. Two signals at 7.33 and 7.29 (*d*, *J* = 8.7 Hz) attribute to H-11 and H-12 signals of the protoberberine D-ring. Similarly, another signal at 7.23 (s) attributes to H-13 of the C-ring. A ring of the protoberberine derivative was represented by signals at 6.72 (s) and 6.3 (s) that correspond to H-1, H-4, H-5, and H-6 signals of the isoquinoline B-ring at 2.90 and 4.31 (2H, *t*, *J* = 6.2 Hz), respectively. Two peaks in the region of 3.96 and 4.03 were observed that represent methoxyl groups. Similarly, signals at 160.10 and 101.38 represent an amide carbonyl and a dioxymethylene group (-OCH_2_O-), respectively (Figure 3). On the basis of the above evidence, compound **1** was found to be an oxyberberine. All signals of oxyberberine were found to be in good agreement with the previous reports [16].

**Figure 4:**
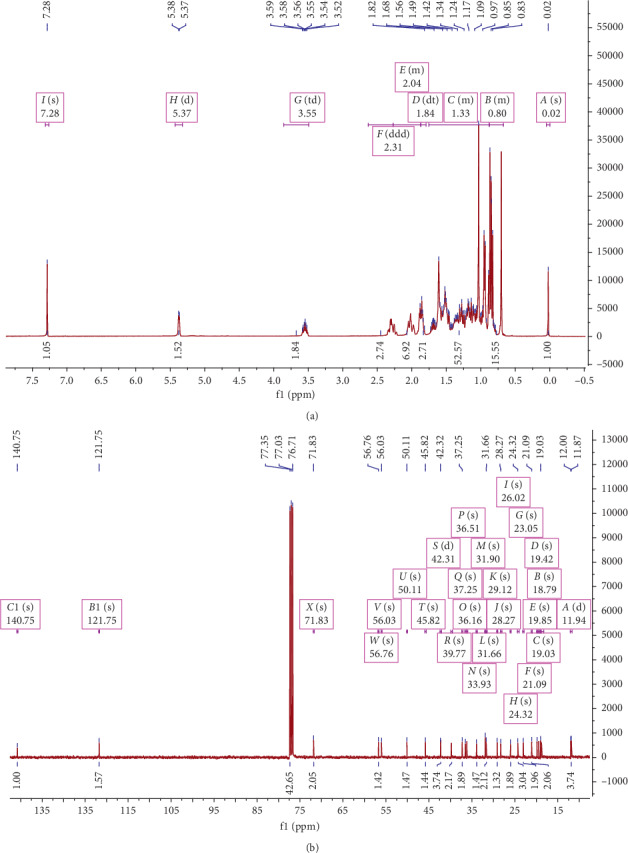
^1^H-NMR (a) and ^13^C-NMR (b) spectra of *β*-sitosterol recorded in CDCl_3_. The chemical shift (d) values are given in ppm with TMS as internal standard, and coupling constants (J) are in Hz.

**Figure 5:**
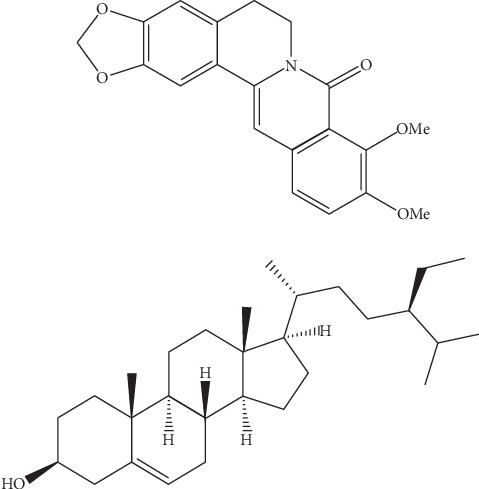
Structures of oxyberberine (**1**) and *β*-sitosterol (**2**) isolated from *Berberis lycium*.

Compound **2** was obtained as white crystals showed complex proton spectra with characteristic phytosterol signals. Two angular methyl singlet signals of 18- and 19-position methyl groups were observed, and the doublet of the 21-, 26-, and 27-position methyl groups was observed on both sides. An olefinic proton (H-6) signal was obtained as a broad doublet at 5.26–5.35 (Figure 4). Combining all data retrieved from signals, the structure was confirmed as *β*-sitosterol [17–19]. Structures of both compounds are shown in Figure 5.

### 3.3. Cytotoxicity of Compounds

**Figure 6:**
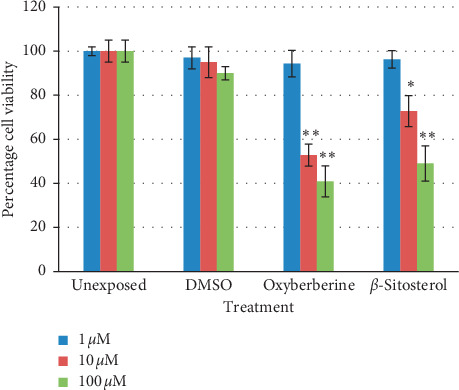
Percentage viability (mean ± SD) of SK-Hep-1 cells at 72-hour treatment against oxyberberine and *β*-sitosterol at different concentrations (1, 10, and 100 *μ*M). ^*∗*^(*P* < 0.01) and ^*∗∗*^(*P* < 0.001) significantly different from unexposed cells (Student's *t*-test).

**Figure 7:**
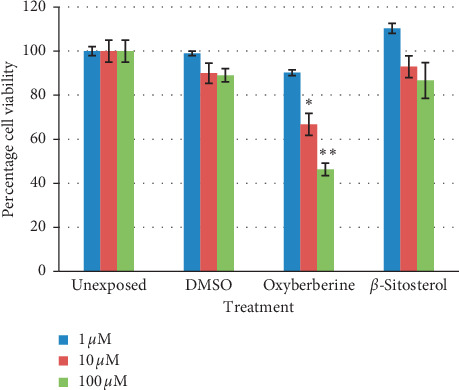
Percentage viability (mean ± SD) of HepG2 cells at 72-hour treatment against oxyberberine and *β*-sitosterol at different concentrations (1, 10, and 100 *μ*M). ^*∗*^(*P* < 0.01) and ^*∗∗*^(*P* < 0.001) significantly different from unexposed cells (Student's *t*-test).

**Figure 8:**
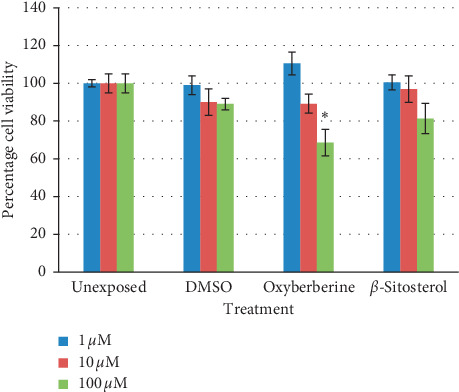
Percentage viability (mean ± SD) of NCI-H1299 cells at 72-hour treatment against oxyberberine and *β*-sitosterol at different concentrations (1, 10, and 100 *μ*M). ^*∗*^(*P* < 0.01) and ^*∗∗*^(*P* < 0.001) significantly different from unexposed cells (Student's *t*-test).

Cytotoxicity of isolated compounds was studied in terms of percent cell viability at 72-hour cultures in SK-Hep-1, HepG2, and NCI-H1299 cells (*n* = 3, mean ± SD). Both oxyberberine and *β*-sitosterol inhibit cell proliferation in a dose-dependent manner. Oxyberberine showed the highest reduction in cell proliferation in SK-Hep-1 cells (IC_50_ = 34.26 ± 3.34 *μ*M), while *β*-sitosterol showed 50 percent reduction at 123.12 ± 3.51 *μ*M. At 100 *μ*M and 10 *μ*M concentrations, oxyberberine reduced viability of SK-Hep-1 cells by 60% and 47% that is significantly different from the unexposed sample at *P* < 0.001 while *β*-sitosterol showed 50.95% reduction in cell viability that is significantly different from the unexposed sample at *P* < 0.001 (Figure 6). In HepG2 cells, oxyberberine also inhibited cell proliferation in a dose-dependent manner. The IC_50_ value was calculated as 62.96 ± 4.12 *μ*M. *β*-Sitosterol showed the IC_50_ value of 140 ± 4.21 *μ*M. HepG2 cells showed 53% reduction in cell viability at 100 *μ*M concentration of oxyberberine that is significantly different from the unexposed sample at *P* < 0.001 while *β*-sitosterol expressed only 17% reduction in cell viability at this concentration (Figure 7). NCI-H1299 cells showed less sensitivity toward oxyberberine with IC_50_ > 600 *μ*M with only 30% reduction in cell viability at 100 *μ*M. There was no significant reduction in cell viability at lower concentrations (1 and 10 *μ*M). NCI-H1299 cells showed no considerable reduction in cell viability with *β*-sitosterol (Figure 8).

## 4. Discussion

In the present study, crude methanolic extract of *Berberis lycium* Royle was partitioned into various polarity-based fractions for the assessment of reduction in cell proliferation. Methanol was used for formation of crude extract because it has the ability to dissolve a wide range of phytochemicals and it can easily degrade the cell wall and exposing interior of cells for the extraction [20]. The *n*-hexane fraction rich in non-polar compounds and chloroform fraction having alkaloids both found active in reducing cell proliferation. Nature of compounds in these cytotoxic fractions was assessed on both normal-phase and reverse-phase thin-layer chromatographies. Good separation of compounds was achieved on normal-phase TLC with petroleum ether and ethyl acetate in combination. A series of normal-phase column chromatographies of cytotoxic extracts of *Berberis lycium* Royle with various combinations of solvents afforded compounds that caused death in SK-Hep-1, HepG2, and NCI-H1299 cells in a concentration-dependent manner.

Oxyberberine was proved to be more cytotoxic than *β*-sitosterol and caused significant reduction in viability of SK-Hep-1 cells as compared to HepG2 and NCI-H1299 cells. As shown in Figure 3, the phenyl ring of oxyberberine is substituent with different groups at C2, C3, C9, and C10. According to a study, significant cytotoxic effect of protoberberine alkaloids was due to the R-O-CH_2_-O-*R*′ (*R* = C2, *R*′ = C3) group on the phenyl ring [21]. The methylenedioxy group between C2 and C3 of the phenyl ring enhances the activity of protoberberine alkaloids in antibacterial and antimalarial bioassays [22]. Thus, the –O-CH_2_-O-functional group at C2 and C3 on the phenyl ring might be a significant influencing factor for the cytotoxic effects of oxyberberine on SK-Hep-1 cells. Previous research conducted on protoberberine alkaloids showed that they interact with different enzymes and nucleic acids and induce apoptosis by inhibiting their functions [23]. NMR spectroscopy provided the data of intercalation of rings C and D in the protoberberine structure with DNA. Berberine, a protoberberine alkaloid, from the genus *Berberis* forms complexes with both DNA and RNA to inhibit oncogene expression [24]. It acts as an inhibitor of N-acetyltransferase (NAT), cyclooxygenase-2 (COX-2), and telomerase. It also regulates the cyclin-dependent kinase (CDK) family of proteins and B-cell lymphoma 2 (Bcl2) family of proteins and caspases [25, 26]. This might be the possible cytotoxic mode of action in treating human cancers.

## 5. Conclusion

Conclusively, two cytotoxic compounds, namely, oxyberberine and *β*-sitosterol, were isolated for the first time from *Berberis lycium* Royle through normal-phase column chromatography and their cytotoxicity was tested against three human cancer cell lines (SK-Hep-1, HepG2, and NCI-H1299). Both of the compounds showed reduction in cell proliferation in a dose-dependent manner. Oxyberberine and *β*-sitosterol showed the highest cytotoxicity in SK-Hep-1 cells while showed moderate cytotoxicity in HepG2 cells. It was also observed that oxyberberine was found to be more cytotoxic against cancer cells than *β*-sitosterol. Both compounds were found less sensitive against NCI-H1299 cells. The cytotoxic potential of *Berberis lycium* Royle extracts and isolated compounds is suggesting that it is a promising candidate for anticancer drug discovery, and further studies should be carried out to trace the mechanism of cytotoxicity of oxyberberine at cellular level.

## Data Availability

The data used to support the findings of this study are included within the article. Graphs of NMR and MS for structure elucidation are available on request.
